# **PET Molecular Imaging Research of Levodopa-Induced Dyskinesias in Parkinson’s Disease**

**DOI:** 10.1007/s11910-017-0794-2

**Published:** 2017-10-03

**Authors:** Gennaro Pagano, Tayyabah Yousaf, Marios Politis

**Affiliations:** Neurodegeneration Imaging Group, Maurice Wohl Clinical Neuroscience Institute, Institute of Psychiatry, Psychology & Neuroscience, King’s College London, 125 Coldharbour Lane, London, Camberwell SE5 9NU UK

**Keywords:** Dyskinesias, Parkinson’s disease, Molecular imaging, Positron emission tomography

## Abstract

**Purpose of Review:**

To review the current status of positron emission tomography (PET) molecular imaging research of levodopa-induced dyskinesias (LIDs) in Parkinson’s disease (PD).

**Recent Findings:**

Recent PET studies have provided robust evidence that LIDs in PD are associated with elevated and fluctuating striatal dopamine synaptic levels, which is a consequence of the imbalance between dopaminergic and serotonergic terminals, with the latter playing a key role in mishandling presynaptic dopamine release. Long-term exposure to levodopa is no longer believed to solely induce LIDs, as studies have highlighted that PD patients who go on to develop LIDs exhibit elevated putaminal dopamine release before the initiation of levodopa treatment, suggesting the involvement of other mechanisms, including altered neuronal firing and abnormal levels of phosphodiesterase 10A.

**Summary:**

Dopaminergic, serotonergic, glutamatergic, adenosinergic and opioid systems and phosphodiesterase 10A levels have been shown to be implicated in the development of LIDs in PD. However, no system may be considered sufficient on its own for the development of LIDs, and the mechanisms underlying LIDs in PD may have a multisystem origin. In line with this notion, future studies should use multimodal PET molecular imaging in the same individuals to shed further light on the different mechanisms underlying the development of LIDs in PD.

## Introduction

More than 50 years since its discovery [1, 2], levodopa remains the gold standard in the management of motor symptoms of Parkinson’s disease (PD) [3]. Although several systems are affected in PD, including dopaminergic, serotonergic, noradrenergic, cholinergic, glutamatergic, opioid and endocannabinoid systems [4], it is the loss of dopamine within the nigrostriatal pathway that leads to the emergence of the cardinal motor symptoms of PD [5]. Exogenous levodopa therapy fundamentally restores synaptic dopamine levels in the striatum, which is essential for the correct execution of movements [6]. However, despite its efficacy, long-term levodopa use is complicated by alterations in motor response, such as the development of levodopa-induced dyskinesias (LIDs) [7]. About 30% of PD patients develop LIDs after only 3 years of levodopa use [8], and approximately 80% of PD patients will develop LIDs over the course of the disease [9, 10].

The mechanisms underlying LIDs are still unclear. Levodopa induces sharp increases in striatal dopamine levels, which are particularly elevated in PD patients who experience LIDs [11]. However, the notion that LIDs are only due to high exposure to levodopa, which progressively lowers the threshold for dyskinesias until the administered concentration of levodopa needed for its antiparkinsonian action will produce LIDs, making the responses inseparable and causing pronounced motor fluctuations between an ‘on’ condition with dyskinesia and an ‘off' condition with severe parkinsonism [12], has been questioned recently [13]. In PD patients who experience LIDs, the administration of a high dose of levodopa induces an antiparkinsonian response and dyskinesias at a comparable threshold. This observation indicates that the therapeutic window between the antiparkinsonian response and dyskinesia does not exist, except for the period before the emergence of dyskinesia, and that also other pathways are needed for the development of LIDs [13]. Another recent study has corroborated this hypothesis [14•]. Elevated putaminal dopamine release was present in de novo PD patients and was associated with an increased risk of later development of LIDs. This suggests that early compensatory changes in striatal dopamine turnover could be a disease-intrinsic predisposing factor for the development of LIDs, maybe due to changes in non-dopaminergic pathways and not related to the long-term exposure to levodopa [14•]. A large number of studies have demonstrated that LIDs rely on a sequence of events, including abnormalities in corticostriatal neurotransmission, postsynaptic changes in proteins and gene expression, altered neuronal firing and plasticity [15, 16].

Molecular imaging modalities are able to identify minimal alterations at the nanomolecular level, and this is a prerequisite to understand subtle changes in brain activity [17, 18]. Positron emission tomography (PET) molecular probes bind a target, such as a receptor, a transporter or an enzyme, with high specificity and power of resolution [17]. PET molecular imaging has revolutionized the possibilities to gain insight into human brain biology and beyond this to understand the physiology and the pathophysiology of neurological diseases [17, 18]. PET radiotracers have provided invaluable insight into the mechanisms underlying LIDs, and have been used to measure dopaminergic [19], serotonergic [20], noradrenergic [21], cholinergic [22], glutamatergic [23], adenosinergic [24], opioid [25] and cannabinoid systems [24], phosphodiesterases [26] and other targets [27].

This review describes the current status of PET molecular imaging of LIDs, and its relation with the underlying mechanisms of PD.

## Presynaptic and Postsynaptic Mechanisms of LIDs

Multiple components of the network between basal nuclei and cortex have been recognized as a substrate for the development of LIDs. At the molecular level, changes in signal transduction and neurotransmission occurring in specific populations of neurons have been linked to the emergence of LIDs [16].

The main component needed for the development of LIDs is a moderate-to-severe loss of dopaminergic terminals in the dorsal putamen, associated with the incapacity of the terminals to store dopamine. In this condition, the same amount of levodopa administered induces higher release of dopamine in the extracellular space [28]. At the same time, in the absence of enough intact dopaminergic terminals, exogenous levodopa is metabolized in other terminals expressing the enzyme aromatic l-amino acid decarboxylase (AADC), such as serotonergic and noradrenergic terminals, which do not possess the molecular machinery to properly control the release of dopamine. This results in higher swings in synaptic levels of dopamine and pulsatile stimulation of postsynaptic receptors located on striatal projection neurons [29].

Impaired presynaptic control of dopamine release from dopaminergic and non-dopaminergic terminals leads to overactivation of striatal dopaminergic receptors. This is a second component also needed for the development of LIDs. Abnormal activation of dopaminergic receptor D_1_ results in hyperactivation of the cyclic adenosine monophosphate (cAMP) signalling pathway [30]. D_1_ receptor-mediated activation of cAMP-dependent protein kinase A (PKA) and of dopamine- and cAMP-regulated protein of 32 kDa (DARPP-32) promotes the stimulation of additional intracellular signalling cascades involved in the regulation of gene transcription and protein synthesis [15]. Overactivation of dopaminergic receptor D_3_ has also been suggested in the development of LIDs [31].

Increased extracellular glutamate concentration [28] and changes in the subcellular distribution of glutamate [32] and adenosine [33] receptor subunits have also been associated with LIDs, together with alterations in the expression of messenger RNA that encodes the precursors of striatal neuropeptides preproenkephalin and preprodynorphin [34].

**Fig. 1:**
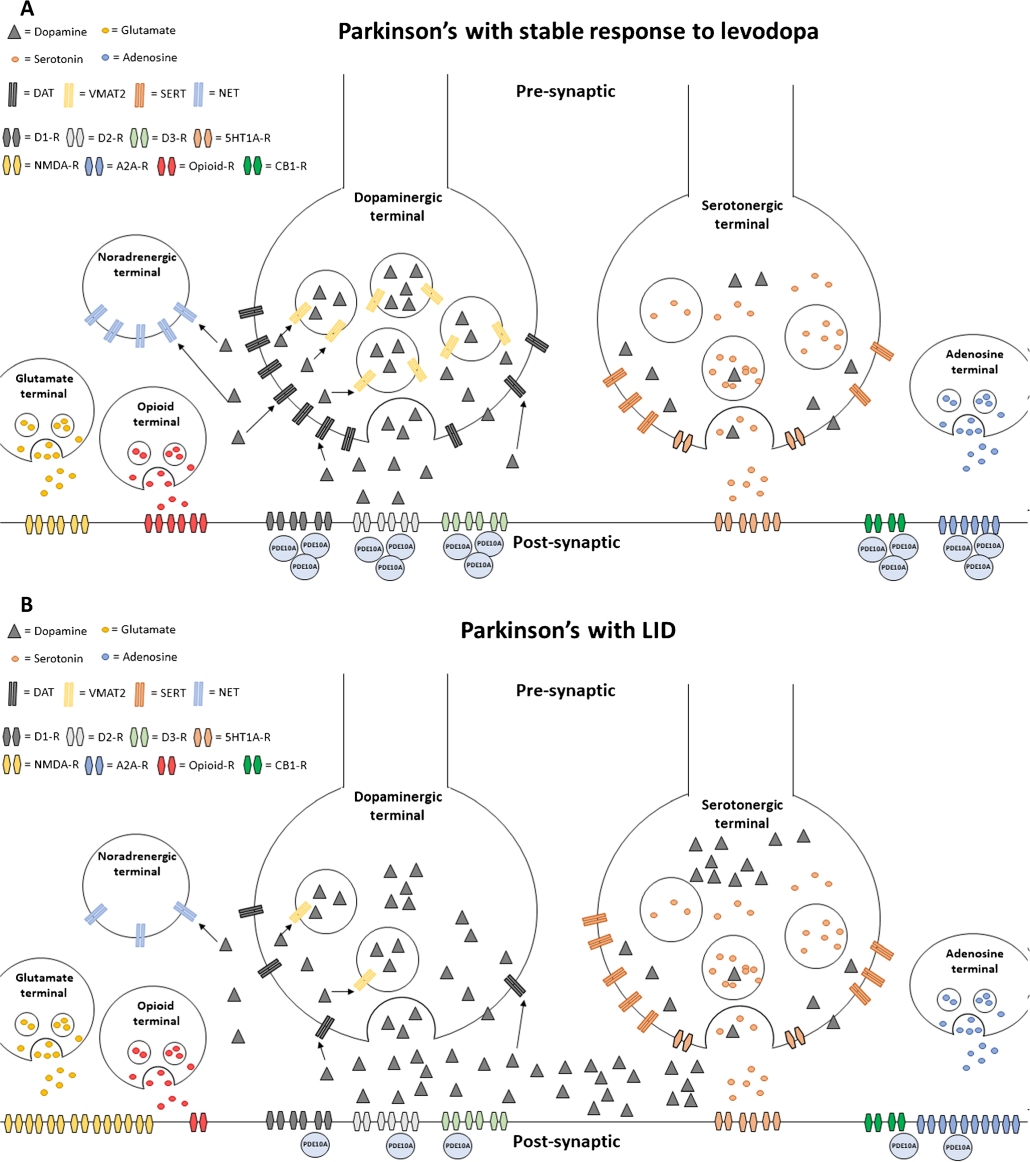
The pathways involved in the development of levodopa-induced dyskinesia (LID). **a** In Parkinson’s disease patients with stable response to levodopa, dopamine transporter (DAT) takes up dopamine and it is stored in the presynaptic vesicles, via vesicular monoamine transporter 2 (VMAT2). As a consequence, dopamine levels remain relatively stable in the synaptic cleft, even after levodopa supplementation. Dopamine levels are maintained within a normal range as serotonergic terminals do not release dopamine excessively after levodopa supplementation, and DAT and noradrenaline transporter (NET) continue to take it up. At the postsynaptic level, the concentrations of dopaminergic, glutamatergic, adenosinergic, opioid and cannabinoid type 1 receptors are within the normal range. However, there is a decline in the concentration of phosphodiesterase 10A (PDE10A), which is an intracellular modulator of these receptors. **b** Parkinson’s disease patients with LIDs lack the capacity to store dopamine, as a result of reduced VMAT2 levels, and fail to take up dopamine, due to the loss of DAT. This is associated with increased dopamine levels in the synaptic cleft. Excessive and inappropriate release of dopamine from serotonergic terminals contributes to the sharp increase of dopamine concentration after levodopa supplementation. At the postsynaptic level, the concentrations of glutamatergic and adenosinergic receptors are increased and those of opioid receptors are reduced. The concentrations of D_1_, D_2_ and cannabinoid type 1 receptors are within the normal range. Compared with Parkinson’s disease patients with stable response to levodopa, there is a further decline of PDE10A concentration, which also correlates with the severity of dyskinesias. A2A-R adenosine A_2A_ receptor, CB1-R cannabinoid receptor type 1, D1-R dopamine receptor D_1_, D2-R dopamine receptor D_2_, D3-R dopamine receptor D_3_, 5HT1A-R serotonin receptor 1A, NMDA-R *N*-methyl-d-aspartate receptor, opioid-R opioid receptor, SERT serotonin transporter

Within the striatum, cAMP signalling is finely regulated by phosphodiesterase 10A (PDE10A). Lesions in nigrostriatal dopaminergic projections in animal models of PD lead to an increase in cAMP levels [35], and treatment with levodopa reduces the high cAMP levels observed in the denervated striatum [35]. The cAMP levels in the corticostriatopallidal pathway are lower at the peak of LIDs compared with those in non-dyskinetic animals, and pretreatment with zaprinast, a non-selective PDE10A inhibitor, prevents the reduction of cAMP levels and reduces the severity of dyskinesias [36]. Altered striatal second-messenger signalling during dyskinesias may be due to the lost ability of striatal neurons to induce both depotentiation and long-term depression [37]. Hence, stimulation of postsynaptic striatal neurons by levodopa-derived dopamine would fail, and dysregulation of PDE10A could be a pathogenic mechanism underlying the dysfunction of second-messenger signalling. We recently demonstrated that PDE10A levels are reduced in the striatum and globus pallidus of PD patients, and are associated with the severity of LIDs [26]. PDE10A might be the final regulator of striatal output, and modulation of its level may be crucial for the control of LIDs. We illustrate these mechanisms in Fig. 1.

## Molecular Imaging of the Dopaminergic System

Dopamine release can be measured by PET with [^11^C]raclopride, a D_2_ receptor antagonist radioligand which competes with endogenous dopamine for D_2_ receptor binding. Changes in D_2_ receptor availability, as reflected by a reduction of baseline [^11^C]raclopride levels after levodopa administration, allows indirect measurement of synaptic dopamine release. PD patients with LIDs showed increased dopamine release after the administration of levodopa compared with PD patients with stable response to levodopa [11, 38]. Greater reduction in putaminal [^11^C]raclopride levels has also been correlated with worse LIDs [39, 40••]. Also, PD patients with LIDs showed lower dopamine levels at 4 h from levodopa challenge, whereas PD patients with stable response to levodopa had unchanged putaminal dopamine levels [38]. This indicates that LIDs are associated with increased and fluctuating synaptic dopamine levels following levodopa administration [11, 38].

PET ligands for the presynaptic dopaminergic system such as [^18^F]fluorodopa ([^18^F]FDOPA), [^18^F]dihydrotetrabenazine, and [^18^F]fluoropropylcarbomethoxyiodophenylnortropane ([^18^F]FP-CIT) or [^11^C]methylphenidate ([^11^C]MP) are reliable tools to assess in vivo striatal AADC activity, the density of vesicular monoamine transporter 2 (VMAT2), and the availability of presynaptic dopamine transporters (DATs). The AADC catalyzes the decarboxylation of levodopa to dopamine, the VMAT2 is the vesicular transporter responsible for the transport of dopamine from the cytoplasm into secretory vesicles and a marker of dopamine storage in the dopaminergic terminals, and the DAT is responsible for the high-affinity uptake of dopamine from the synaptic cleft, respectively [41].

Putaminal AADC activity, measured by [^18^F]FDOPA PET, is lower in PD patients, and correlates with the severity of motor symptom rigidity and bradykinesia [42]. A retrospective study was recently performed in 31 de novo PD patients who underwent quantitative [^18^F]FDOPA PET to measure the effective distribution volume ratio, as a marker of dopamine release, and who were followed up to the development of LIDs [14•]. During a mean 7-year follow-up period, 35.5% (11/31) developed LIDs. They had lower baseline effective distribution volume ratios in the putamen than those who did not develop LIDs, indicating higher dopamine release at the baseline, independently of the use of levodopa. PD patients with LIDs showed 12% reduction in caudate and 28% reduction in putaminal [^18^F]FDOPA uptake compared with PD patients with a stable response to levodopa [43]. At early stages of PD, increased uptake of [^18^F]FDOPA in the serotonergic and noradrenergic terminals, which possibly reflects a compensatory upregulation of AADC, has also been shown, and in the globus pallidus internal segment, which possibly reflects dopamine storage capacity [44]. A recent study with [^18^F]dihydrotetrabenazine showed that in advanced PD patients, lower VMAT2 density in the caudate, putamen and substantia nigra was correlated with the severity of motor complications [45]. The increase of [^18^F]FDOPA uptake in the globus pallidus internal segment is lost with the progression of the disease in advanced PD patients with LIDs [44]. This may indicate that a loss of dopamine storage and capacity in the globus pallidus internal segment is also important for the development of LIDs. The role of the globus pallidus in the development of LIDs has also been confirmed at the postsynaptic level [46]. PD patients with LIDs showed higher D_3_ receptor levels in the globus pallidus, measured with 4-[^11^C]propyl-9-hydroxynaphthoxazine PET, compared with PD patients with stable response to levodopa [46]. This might reflect a D_3_ receptor upregulation in the globus pallidus and, together with the incapacity to store dopamine, the non-ability to sustain a normal pattern of pallidal output to motor cortex.

The presence of moderate-to-severe presynaptic dopaminergic terminal loss seems to play a role also in the development of LIDs, inducing an uncontrolled dopamine release [47]. A retrospective study conducted in 127 de novo patients with PD, who underwent [^18^F]FP-CIT PET at the time of diagnosis, showed that lower putaminal DAT levels at baseline was a predictor of the development of LIDs at the 3-year follow-up. A study with [^11^C]MP PET showed that advanced PD patients with motor fluctuations and LIDs had reduced DAT expression in the putamen compared with advanced PD patients with motor fluctuations but no LIDs [48]. Integrity of DAT function is central in the regulation of dopamine levels in the synaptic cleft, and a gradual loss of DAT availability as PD progresses results in the loss of compensatory reuptake needed when dopamine levels substantially rise after a dose of levodopa [48].

LIDs are unlikely to result from alterations in striatal D_1_ or D_2_ receptor availability. PD patients with LIDs and PD patients without LIDs showed no differences in striatal D_1_ and D_2_ receptor availability, as assessed with [^11^C]SCH23390 PET and [^11^C]raclopride PET, respectively [49].

This evidence is in support of the concept that an increased and fluctuating dopamine release is necessary but not sufficient for the development of LIDs, and other non-dopaminergic pathways may play a role.

## Molecular Imaging of the Serotonergic System

Serotonergic terminals express AADC, and are able to transform exogenous levodopa into dopamine [50]. PET ligands for presynaptic serotonergic transporter (SERT) such as *N*,*N*-[^11^C]dimethyl-2-(2-amino-4-cyanophenylthio)benzylamine ([^11^C]DASB) are reliable tools to assess in vivo serotonergic terminals [51]. The serotonergic system is profoundly affected in PD, and its role in the development of LIDs has been evaluated in a recent meta-analysis [52•] and in several PET studies [40••, 53–55]. PD patients with LIDs showed a relative preservation of serotonergic terminals in the putamen, but the administration of the same levodopa dose induced markedly higher striatal synaptic dopamine release, evaluated with [^11^C]raclopride challenge, compared with PD patients with stable responses to levodopa. Oral administration of the serotonin receptor type 1A agonist buspirone, a presynaptic modulator of the serotonergic system, before levodopa administration reduced levodopa-evoked striatal synaptic dopamine release and attenuated LIDs [40••]. Another study showed that PD patients with LIDs had no difference in striatal dopaminergic disease, assessed by [^18^F]FP-CIT PET, compared with non-dyskinetic PD patients; however, the [^11^C]DASB to [^18^F]FP-CIT binding ratio (indicating serotoninergic to dopaminergic terminal availability) was higher in the putamen of PD patients with LIDs [53]. These findings were confirmed by another study, which demonstrated that the SERT to DAT ratio increases as PD progresses and patients experience LIDs [54]. Overall, these findings suggest that as the dopaminergic innervation in the striatum becomes critically low, the serotonergic system plays an important role in development of LIDs by handling dopamine synaptic levels in an unregulated manner. Moreover, the role of the globus pallidus in the development of LIDs has been demonstrated also with [^11^C]DASB PET. PD patients with LIDs had relative preservation of serotonergic terminals in the globus pallidus and increased pallidal dopamine release, measured with levodopa challenge and PET [55]. Serotonergic mechanisms such as excessive striatal innervation and high serotonin to dopamine striatal terminal ratio have also been associated with the development of graft-induced dyskinesias in PD patients who underwent striatal transplantation with fetal ventral mesencephalic tissue [56–58]. These findings support the role of serotonergic terminals in the aberrant release of striatal dopamine and in promoting the development of dyskinesias in patients with PD.

## Molecular Imaging of the Noradrenergic System

The main presynaptic component of noradrenergic pathways is from the locus coeruleus projecting to the forebrain, including the striatum [59]. As for the serotonergic terminals, the noradrenergic terminals express AADC and are able to convert levodopa into dopamine [60]. Increased release of dopamine via noradrenergic terminals may promote dysregulated striatal dopamine levels and, consequently, LIDs [61]. A recent randomized clinical trial showed that fipamezole, a selective α_2_-adrenergic receptor antagonist, was able to reduce LIDs without exacerbating parkinsonism [62]. Unfortunately, PET ligands for noradrenaline transporter such as 2-(α-(2-[^11^C]methoxyphenoxy)benzyl)morpholine or [^11^C]methyl reboxetine are not reliable tools to assess in vivo noradrenaline transporter activity [63], showing very low signal in the striatum. The development of selective PET radioligands tagging the noradrenergic system is warranted to better understand its involvement in the development of LIDs.

## Molecular Imaging of the Cholinergic System

The main presynaptic component of cholinergic pathways is from the pedunculopontine nucleus pars compacta, projecting to the forebrain as well as various subcortical structures such as the thalamus [64]. *Post-mortem* studies in humans have shown decreases in acetylcholinesterase activity in PD patients with and without dementia [65]. PET ligands for the presynaptic cholinergic system such as *N*-[^11^C]methyl-4-piperidyl acetate ([^11^C]MP4A) and *N*-[^11^C]methylpiperidinyl propionate ([^11^C]PMP) are reliable tools to assess in vivo acetylcholinesterase activity [66]. PET studies using [^11^C]MP4A and [^11^C]PMP have demonstrated 11–12% decreases in cortical and subcortical acetylcholinesterase activity in PD patients without dementia [67, 68]. No PET studies investigating the role of acetylcholinesterase in the development of LIDs have been performed in PD patients; thus, this component needs further investigation.

The main postsynaptic component of cholinergic pathways is from cholinergic neurons intrinsic to the striatum [69]. Global striatal output is significantly influenced by the cholinergic interneurons [70]. They represent less than 5% of the total striatal neurons but provide a major contribution to the release of dopamine [71]. In preclinical studies, elevated cholinergic signalling has been associated with LIDs [72], and muscarinic and nicotinic receptor antagonists partially attenuated the expression of LIDs [73, 74]. LIDs may be attenuated by the ablation of the striatal cholinergic interneurons, without affecting the beneficial antiparkinsonian effect of levodopa [75]. *Post-mortem* studies in humans showed reduced levels of muscarinic and nicotinic receptors in the striatum of PD patients [76, 77]. This potentially suggests a downregulation of the receptors induced by increased cholinergic signalling. PET ligands for the postsynaptic cholinergic system such as [^18^F]A-85380 and *N*-[^11^C]methylpiperidyl benzilate ([^11^C]NMPB) are reliable tools to assess in vivo nicotinic and muscarinic receptors respectively. A PET study using [^18^F]A-85380 was performed in PD patients and showed reduced levels of nicotinic receptors in the striatum and substantia nigra compared with the levels in controls [78]. There were no associations between [^18^F]A-85380 levels and disease severity, but LIDs have not been evaluated in detail. No PET studies have been performed in humans investigating the role of the cholinergic system in LIDs with [^18^F]A-85380, [^11^C]NMPB or other tracers; thus, this component needs further investigation.

## Molecular Imaging of the Glutamatergic System

Glutamate is an excitatory neurotransmitter that acts through glutamate *N*-methyl-d-aspartate (NMDA) receptors, which include the NR1, NR2A and NR2B subtypes [79]. Experimental studies have shown that hyperphosphorylation of these subunits is associated with increased glutamatergic neurotransmission and the development of LIDs [80]. PET ligands for NMDA receptors such as [^11^C]CNS51619 are reliable tools to investigate in vivo the glutamatergic system [81]. Only one PET study investigating the glutamatergic system has been performed in PD patients with LIDs [81]. PD patients not receiving medication had no differences in the basal nuclei and in the motor cortex compared with PD patients with stable response to levodopa. However, PD patients with LIDs receiving medication showed higher [^11^C]CNS51619 uptake in the caudate, putamen and precentral gyrus compared with PD patients without LIDs, suggesting that dyskinetic patients may have abnormal glutamatergic transmission in motor areas following levodopa administration [81]. These findings support the hypothesis that glutamate transmission is important in the development of LIDs, and provide the physiological basis of why amantadine, a non-competitive antagonist of the NMDA receptor, is currently the most effective treatment for LIDs [82].

## Molecular Imaging of the Adenosinergic System

Adenosine is an endogenous ligand for four receptor subtypes: A_1_, A_2A_, A_2B_ and A_3_ [89]. The adenosine A_2A_ receptors are expressed in the striatum and interact with the dopamine D_2_ receptor function, via the cAMP pathway [83]. PET ligands for the adenosinergic system such as [1-*methyl-* ^11^C]8-dicyclopropylmethyl-1-methyl-3-propylxanthine and [7-*methy*l-^11^C]-(*E*)-8-(3,4,5-trimethoxystyryl)-1,3,7-trimethylxanthine or [^11^C]SCH442,416 are reliable tools to measure in vivo A_1A_ and A_2A_ receptors, respectively. Two PET studies investigating the adenosinergic system have been performed in PD patients with LIDs [84, 85]. They both showed increased striatal adenosine A_2A_ receptor availability in PD patients with LIDs [84, 85]. A_2A_ receptor binding sites could serve as potential pharmacological targets for the management of LIDs. A recent randomized clinical trial in PD patients with LIDs showed that use of KW-6002, a selective adenosine A_2A_ receptor antagonist, was effective in alleviating this motor complication [86].

## Molecular Imaging of the Opioid System

Three opioid receptors subtypes (μ, κ and δ) are involved in regulating dopamine functions [87]. [^11^C]diprenorphine, a non-selective opioid receptor PET ligand, is a reliable tool to assess in vivo the opioid system [25]. The main limitation of this tracer is that it binds all three opioid receptor subtypes with similar affinity. One PET study has been performed with [^11^C]diprenorphine in PD patients, and showed reduced striatal, thalamic and cingulate opioid receptor binding and increased prefrontal opioid receptor binding in PD patients with LIDs compared with PD patients with stable response to levodopa [88]. No correlations were found between [^11^C]diprenorphine levels and PD severity, disease duration, or duration of levodopa treatment [88]. Further PET studies using more selective opioid radioligands could provide better insight into the role of the opioid system in the pathophysiology of LIDs.

## Molecular Imaging of the Cannabinoid System

The cannabinoid type 1 (CB1) receptor is a seven transmembrane G-protein-linked receptor located predominantly in the striatal medium spiny neurons [89]. CB1 receptors inhibit adenylate cyclase by interacting with G_i/o_ proteins in the direct pathway, and they counteract D_2_ inhibition of adenylate cyclase coupling with G_s_ proteins in the indirect pathway, thus having a key role in the control of movement [90]. Alterations in the availability of the cannabinoid receptors have also been related to the development of LIDs [91]. PET ligands such as [^18^F]MK-9470 are a reliable tool to measure in vivo CB1 receptors [92]. One study has been performed in PD patients, using [^18^F]MK-9470, which demonstrated a significant decrease in CB1 receptor availability in the substantia nigra compared with healthy controls. No correlation was found between reductions in CB1 receptor levels and the severity of LIDs [92]. Further PET studies with highly selective CB1 receptor radioligands, such as (3*R*,5*R*)-5-(3-[^11^C]methoxyphenyl)-3-((*R*)-1-phenylethylamino)-1-(4-trifluoromethylphenyl)pyrrolidin-2-one [93], are needed to clarify the role of the cannabinoid system in the development of LIDs.

## Molecular Imaging of PDE10A

PDE10A is mainly expressed in striatal medium spiny neurons, where it regulates the cAMP/PKA/DARPP-32 signalling cascade, thus plays a key role in the regulation of the global striatal output and in promoting neuronal survival [94, 95]. PDE10A has received increased attention since the observation that its pharmacological inhibition in a PD animal model significantly reduced motor symptoms and LIDs [96]. The PET ligand [^11^C]IMA107 has been used to quantify PDE10A expression in vivo in moderate–advanced PD patients [26]. PD patients showed lower PDE10A levels in the caudate, putamen and globus pallidus compared with healthy controls. Higher Unified Dyskinesia Rating Scale scores in PD patients with LIDs correlated with lower PDE10A levels in the caudate and putamen [26]. This provides evidence for the role of PDE10A within the caudate and putamen in the development of LIDs in PD. Currently, a clinical trial (NCT02687542) is evaluating the effect of PDE10A inhibitors on motor complications in advanced PD patients.

## Conclusions

Studies have provided robust evidence that LIDs in PD are associated with elevated and fluctuating striatal dopamine synaptic levels arising from an imbalanced dopaminergic to serotonergic terminal ratio, with the latter playing a key role in mishandling presynaptic dopamine release. However, other non-dopaminergic systems and pathways such as the glutamatergic, adenosinergic and opioid systems and phosphodiesterase 10A may play important roles in the development of LIDs in patients with PD. The implementation of novel PET ligands is warranted to unveil unexplored mechanisms of underlying pathophysiology of LIDs, and applications of multimodal PET molecular imaging approaches combining different tracers may shed further light on the mechanisms underlying the development of LIDs in PD.
